# A rare case of pancreatic adenocarcinoma accompanied by venous thrombosis, pleural and pericardial effusions

**DOI:** 10.1097/MS9.0000000000001870

**Published:** 2024-02-28

**Authors:** Husam Shawakh, Hassan Bdeiwi, Rashed Aljundi, Fateh Kashkash, Abdullah Khoury

**Affiliations:** a University of Aleppo, Faculty of Medicine; b Department of Pulmonology, Aleppo University Hospital, University of Aleppo, Faculty of Medicine, Aleppo, Syria

**Keywords:** case report, pancreatic adenocarcinoma, pericardial effusions, pleural effusions, venous thrombosis

## Abstract

**Introduction:**

Pancreatic cancer is a deadly type of cancer with few symptoms until metastasis. It poses a high risk of cancer-associated thrombosis.

**Case presentation:**

A 73-year-old male presented with fatigue, shortness of breath, weight loss since 9 months, and blood clots recently in his legs. Chest radiography revealed fluid accumulation in pleural and pericardial cavities. Later, a fluid examination revealed the presence of malignant cells in the pericardial fluid. After immunological tests and an upper gastrointestinal endoscopy were performed, a pancreatic tumour was suspected. The patient was administered anticoagulant treatment and palliative care, which resulted in improvement after one month.

**Discussion:**

Pancreatic adenocarcinoma is a highly aggressive cancer with a strong tendency to metastasize, leading to pericardial and pleural effusion, thrombophlebitis, and poor prognosis.

**Conclusion:**

This case indicates that venous thrombosis, pleural and pericardial effusions could be symptoms related to a pancreatic tumour.

## Introduction

Highlights

- The trio of pericardial effusion, thrombophlebitis, and pleural effusion can be indicative of pancreatic cancer
- The fact that the pleural fluid is free of tumour cells does not rule out the spread of the tumour in other body fluids.
- Also the tumour is stage 4, It is possible to find malignant cells in the pericardial fluid only and not in the pleural fluid.

Pancreatic cancer, which accounts for only 2% of all cancers, is one of the deadliest types of cancers, causing 5% of cancer-related deaths. During the development and progression of pancreatic cancer, most patients do not experience any symptoms until they develop metastasis. The most common type of pancreatic cancer is pancreatic ductal adenocarcinoma (PDAC)^1^.

The symptoms of PDAC can vary and include weight loss, abdominal pain, jaundice, and anorexia. Patients with pancreatic cancer may experience gastrointestinal bleeding as a complication^2^.

Malignancy of the pancreas is a well-known cause of clinically significant vascular thrombosis. This can lead to a 7–28 times higher risk of venous thromboembolism across all types of cancers. Pancreatic cancer is one of the malignancies that poses the highest risk of cancer-associated thrombosis (CAT) among all cancers^3^.

We report here a rare case of pancreatic cancer accompanied by arterial and venous thrombosis, pericardial effusion, and pleural effusion. These findings are rare to present together and to be representative of a pancreatic cancer.

## Case presentation

A 73-year-old male smoker with no prior medical issues arrived at the emergency room with complaints of fatigue, shortness of breath, and cough. The patient reported significant weight loss within the last 9 months. Upon clinical examination, the patient’s blood pressure was 110/70 mmHg, SPO_2_ was 89–90%, obvious jugular vein congestion was observed, and respiratory sounds on the right side of the chest were faint. Abdominal tenderness was observed below the right costal margin. The right foot also showed oedema and cyanosis.

Doppler ultrasound of the lower extremity revealed the presence of blood clots in the left popliteal vein, extending to a portion of the lower third of the left superficial femoral vein, causing complete blockage. Similarly, thrombi were observed in the right tibiofibular trunk, causing complete blockage, extending to a portion of the lower third of the left superficial femoral vein. In addition, atheromas were identified in the posterior walls of both the left and right common femoral arteries, causing 44% narrowing in the right artery and 41% narrowing in the left artery. These findings result in reduced blood flow to the lower extremities.

**Figure 1:**
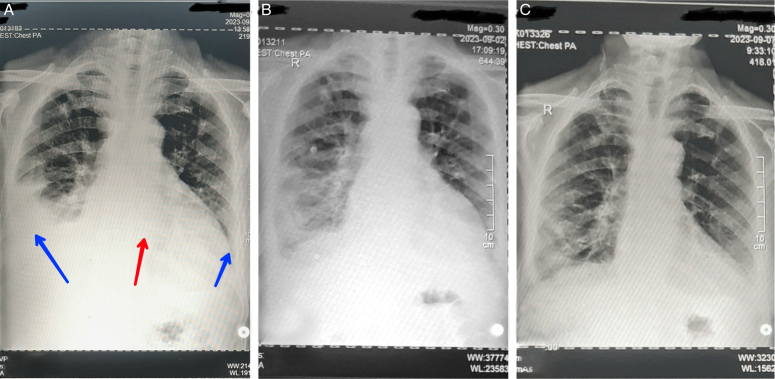
Chest X-ray. (A) Pleural effusion in both lungs, with a more pronounced loss of the costo-phrenic angle in the right lung (blue arrow). Additionally, there is pericardial effusion, indicated by a spherical enlargement of the cardiac shadow, resembling a water bottle (red arrow). (B) The day after pleural aspiration. (C) One week after pleural and pericardial fluid aspiration.

Chest radiography revealed a serious accumulation of fluid in the right pleural and pericardial cavities. Both fluids were drained and sent to the pathology department for testing. The results showed that there were no malignant cells in the pleural fluid; however, they were detected in the pericardial fluid (Fig. 1).

**Figure 2:**
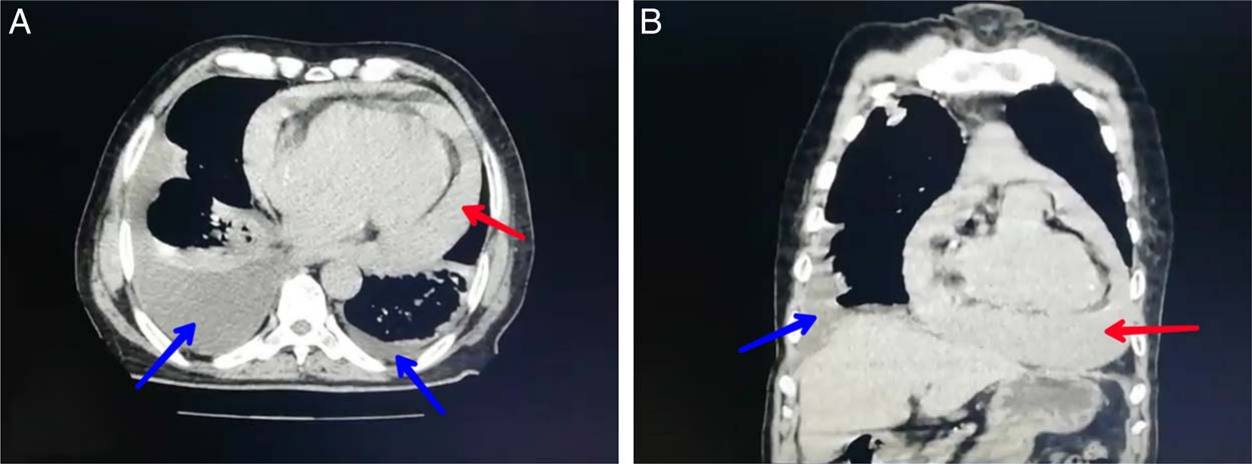
CT (A) shows a cross section, while (B) shows a coronal section. Both sections detect a pleural effusion (indicated by the blue arrow) and a pericardial effusion (indicated by the red arrow).

Chest computed tomography (CT) showed pleural and pericardial effusion ( Figure 2).

After analyzing the pericardial fluid that had been processed by centrifugation, it was observed that the cellular density was moderate, primarily consisting of a large number of transitional epidermal germinal cells. The size of the cells varied moderately, with rudimentary cytoplasm and rare vacuolation. The nuclei are mostly large but occasionally located laterally and possess irregular edges. These cells are rich in chromatin and exhibit active mitotic activity. In addition, reactive inflammatory cells can be seen scattered among tumour cells.

Examination of the fluorescent immunological markers showed positive results for CK7, whereas calretinin, p63, TTF1, HMB54, CK20, CDx2, and PSA tested negative. This suggests that the tumour cells could have originated from either the pancreas or the stomach.

Further tests ruled out a gastric tumour through upper gastrointestinal endoscopy. Therefore, the patient was diagnosed with pancreatic adenocarcinoma.

Laboratory tests also revealed the following results: leucocytes, 22 200/mm^3^; neutrophils, 84.7%; lymphocytes, 9.5%, C-reactive protein (CRP) 27 mg/dl, fasting glucose 237 mg/dl, urea 147 mg/dl, and creatinine 1.78 mg/dl, carcinoembryonic antigen (CEA) 27 ng/ml, CA19-9 55.9 U/ml, prothrombin time 25.3 sec.; activity 34,5%, International Normalized Ratio 2.02, Alkaline Phosphatase 166 U/l, total bilirubin 1.8 mg/dl; and direct bilirubin, 1 mg/dl.

The patient was administered anticoagulant medication and received palliative care. A CT scan with contrast was not conducted because of the patient’s clinical condition and elevated levels of kidney function indicators such as creatinine and urea. Moreover, abdominal MRI could not be performed because the patient refused to undergo the procedure. Abdominal ultrasonography did not reveal any pathological findings, and we were unable to detect the pancreas.

Follow-up after a month revealed improvement in symptoms.

## Discussion

Pancreatic adenocarcinoma is a highly aggressive malignancy, which is known for its strong tendency to metastasize, contributing to the poor prognosis associated with it risk factors for pancreatic cancer include tobacco use, diabetes mellitus, obesity, chronic pancreatitis, advanced age, male sex, diabetes mellitus, obesity^4^.

Cancers, such as lung cancer, breast cancer, melanoma, and lymphoma, tend to spread to the pericardium and heart, resulting in conditions such as carcinomatous pericarditis and pericardial effusion. Pericardial effusion caused by metastatic spread can occur through various mechanisms, including elevated hydrostatic pressure, cachexia-induced hypoalbuminemia, and direct implantation of tumour cells. These mechanisms can lead to fluid accumulation in the pericardial space, potentially causing cardiac tamponade^5^.

Several studies have demonstrated that primary gastrointestinal tumours can cause metastatic pericardial effusion through direct extension, hematogenous dissemination, and lymphatic spread. Malignant pericardial effusion is linked to poor outcomes, and we are not aware of any cases where patients with pancreatic cancer got intrapericardial treatment^6^. Individuals with pericardial effusion may experience symptoms such as dyspnoea, hypotension, tachycardia, cold sweats, and fatigue. If these symptoms arise, echocardiography is highly recommended as it is the most effective diagnostic method for detecting pericardial effusion and identifying signs of cardiac tamponade. Although tamponade diagnosis is generally based on clinical criteria, echocardiography can deliver a precise and prompt diagnosis of this hazardous condition^7^.

Our patient had cardiac tamponade and thrombophlebitis. Studies have revealed that this phenomenon is prevalent among various tumour types; however, pancreatic carcinoma is more frequently associated with it than documented in the tumour Registry data. Notably, tumours located in the body and tail of the pancreas have a higher incidence of thrombophlebitis than those located in the head^3^.

This observation may be attributed to the correlation between trypsin and plasma antithrombin levels^8^. It is worth highlighting that adenocarcinoma, particularly when affecting the lung or pancreas, is frequently associated with deep vein thrombosis (DVT) due to the increased expression of procoagulants in these cancer cells. Biochemical changes triggered by cancer can result in thrombophilia or malignancy, a condition that promotes blood clotting and can result in various clinical syndromes. Moreover, factors such as immobility, pressure, and chemotherapy-induced endothelial damage can further increase the risk of blood clotting. Venous thrombosis is the most prevalent clinical outcome leading to DVT or pulmonary embolism^9^.

Pleural effusion frequently arises as a complication of pancreatic cancer, resulting in an abnormal accumulation of fluid within the pleural cavity surrounding the lungs, which is typically indicative of advanced disease and a less favourable prognosis. The underlying causes may include the spread of tumour cells to the pleura or obstruction of lymphatic vessels near cancerous lesions^10^.

## Conclusion

Our patient exhibited a trio of uncommon complications related to pancreatic cancer, including thrombophlebitis, pericardial, and pleural effusions. The diagnosis was made only after thorough investigations that included an examination of pericardial fluid and the presence of tumour cells with positive CK7 detected through fluorescent immunological markers, which served as a crucial diagnostic clue. This case underscores the importance of considering pancreatic tumours as a possible cause of such clinical presentations.

## Ethics approval and consent to participate

The patient was informed of the availability and importance of the data, including clinical data, images, and health information, as described in this article.

## Consent for publication

Written informed consent was obtained from the patient for the publication of this case report and any accompanying images.

## Source of funding

The authors declare no sources of funding for this manuscript from any organization or institution.

## Author contribution

F.K. supervised the writing of the manuscript. H.B. and R.A. wrote the manuscript. H.S. contributed to writing and correspondence to this manuscript. All authors have read and approved the final manuscript.

## Conflicts of interest disclosure

The authors declare no conflicts of interest.

## Research registration unique identifying number (UIN)

None.

## Guarantor

Fateh Kashkash.

## Availability of data and materials

All data underlying the results are available as part of the article and no additional source data are required.

## Provenance and peer review

Not commissioned, externally peer-reviewed.
